# Augmented reality in medical education?

**DOI:** 10.1007/s40037-013-0107-7

**Published:** 2014-01-25

**Authors:** Carolien Kamphuis, Esther Barsom, Marlies Schijven, Noor Christoph

**Affiliations:** 1 Department for Evaluation, Quality and Development of Medical Education, Radboud University Nijmegen Medical Center, 306 IWOO, PO Box 9101, 6500 HB Nijmegen, the Netherlands; 2 Department of Surgery, Academic Medical Center, PO Box 22660, 1100 AD Amsterdam, the Netherlands; 3 Center for Evidence-Based-Education, Academic Medical Center, PO Box 22660, 1100 AD Amsterdam, the Netherlands

**Keywords:** Augmented reality, Technology enhanced learning, Medical applications, Transfer of learning

## Abstract

Learning in the medical domain is to a large extent workplace learning and involves mastery of complex skills that require performance up to professional standards in the work environment. Since training in this real-life context is not always possible for reasons of safety, costs, or didactics, alternative ways are needed to achieve clinical excellence. Educational technology and more specifically augmented reality (AR) has the potential to offer a highly realistic situated learning experience supportive of complex medical learning and transfer. AR is a technology that adds virtual content to the physical real world, thereby augmenting the perception of reality. Three examples of dedicated AR learning environments for the medical domain are described. Five types of research questions are identified that may guide empirical research into the effects of these learning environments. Up to now, empirical research mainly appears to focus on the development, usability and initial implementation of AR for learning. Limited review results reflect the motivational value of AR, its potential for training psychomotor skills and the capacity to visualize the invisible, possibly leading to enhanced conceptual understanding of complex causality.

## Introduction

The medical domain is a domain in which complex learning occurs [1, 2]. Complex learning involves understanding complex physiological systems, developing adaptive expertise and acquiring the collaborative skills required in multidisciplinary medical practice. It involves mastery of competencies that enable the individual to effectively perform occupational activities to the standards expected in the professional environment. This requires ample opportunity to practice and the ability to experience all possible variations in contexts and circumstances in order to reach the expert level.

Learning in the medical domain is to a large extent workplace learning, from undergraduate clerkships to postgraduate residency training. However, learning in workplace settings is sometimes too risky, difficult to organize, time-consuming and/or expensive. The complexity of the work environment may also be daunting for the trainee. Excellence in the professional context therefore requires an appropriate preparation of the trainee in a dedicated training setting. This training setting should enable transfer of learning: the application of competencies acquired in medical training into the professional workplace.

Meaningful learning is a prerequisite for transfer of learning to occur [3]. Meaningful learning is [4]:

- active: it requires interaction with the world; ‘learning by doing’
- constructive: it requires integrating new experiences within the existing knowledge
- intentional: it requires goal-directed behaviour
- authentic: it requires the use of real-world tasks with adapted complexity within a realistic environment
- cooperative: it requires communication and collaboration.

Recent instructional theories tend to focus on whole-task training [2] in order to achieve meaningful learning and transfer. Whole-task training refers to the practice of more and more complex versions of whole, complex cognitive skills in a realistic and authentic training task. ‘Whole’ refers to the complete view of the whole skill (with underlying constituent skills) as is required for professional performance in the real world. Appropriate sequence of learning tasks and scaffolds are needed to promote systematic acquisition and integration of competencies.

Educational technology has the potential to offer a safe, suitable and cost-effective training setting in which whole, real-world training tasks can be practised. In such controlled environments, learners can make errors without adverse consequences, while instructors can focus on learners rather than patients. Those learning environments also give learners opportunities for just-in-time and just-in-place learning.

The annual HORIZON report describes emerging technologies that are likely to have an impact on teaching and learning in higher education. Innovative technologies mentioned in these reports are for instance, game-based learning, learning analytics, mobile learning, electronic books and open educational resources. In the 2010 and 2011 reports also augmented reality (AR) is referred to as a promising technology for education [5, 6]. ‘*Augmented reality has strong potential to provide both powerful contextual,* in situ *learning experiences and serendipitous exploration and discovery of the connected nature of information in the real world.*’ [6, p. 22].

Educational technology, and more specifically AR, is promising for facilitating meaningful learning and transfer; furthermore it may offer organizational advantages because:

- the physical training environment may be very similar to, if not the same, as the professional work environment
- the augmented (virtual) part may visualize the invisible and simulate relevant 3D [7], tactile and other aspects of the real world task
- the AR learning environment may provide the necessary variations in the training task including collaboration which supports authentic learning
- the real time interactive nature of AR provides immediate learner feedback which supports taking control over the learning process
- AR learning environments do not always require an expert or instructor to observe trainee performance
- AR learning environments can provide situated just-in-time and just-in-place learning.

This article aims at describing a few implementations of AR training systems for typical medical learning tasks in order to highlight its potential for complex learning in this domain. In order to do that, first the concept of AR is explained in more depth and some technical background is given. Then, two AR learning systems for visualizing parts of the human body are described and the application of AR for training laparoscopy skills is discussed. Finally, empirical results on the usefulness and effectiveness of AR systems are discussed and waypoints for further research are given.

## What is augmented reality and how does it work?

AR can be regarded as a technology that integrates computer-generated objects and/or virtual content into the real world, thereby enhancing the perception of reality [8]. Azuma et al. [9, 10] provide three commonly accepted criteria that denote AR systems as systems that [11]

1. Combine real and virtual
2. Are interactive in real time
3. Register in three dimensions.

**Box 1:** Milgram’s reality–virtuality continuum [12]

Milgram, Takemura, Utsumi, and Kishino [12] place AR in between reality (real environment) and virtuality (virtual environment) on the reality-virtuality continuum. This is a continuous scale ranging between reality, where everything is physical, and virtual reality, where a complete virtual environment is created by a computer. Mixed reality is located between them, and includes augmented reality (AR) and augmented virtuality (AV)	

So, the fundamental idea of AR is to combine or mix the view of the real environment with additional virtual content. This virtual content can appeal to different senses such as sight, hearing, touch and smell [10, 13].

To connect virtual content to the real world, a computer device is needed. This device provides a window (display) through which the physical world can be seen. For the virtual components to become visible in this window, as an *augmentation* to reality, a software application on this device is needed as well.

There are many different hardware devices that can be used for AR. The most commonly used is a handheld device like a smartphone or a tablet. A non-handheld device is a *Head Mounted Display* (HMD). The display is worn on the user’s head, mounted in a helmet or a pair of glasses. The advantage of the HMD is that the display stays in front of the eyes, no matter in what direction the user might look, supporting situation awareness. Google Glass is an example of an HMD used by the Radboud University Medical Center, Nijmegen and the Academic Medical Center, Amsterdam to explore the possible added value for health care and medical education. All hardware devices used for AR have in common that they have a processor, a camera, GPS, sensors and/or a compass.

**Fig. 1:**
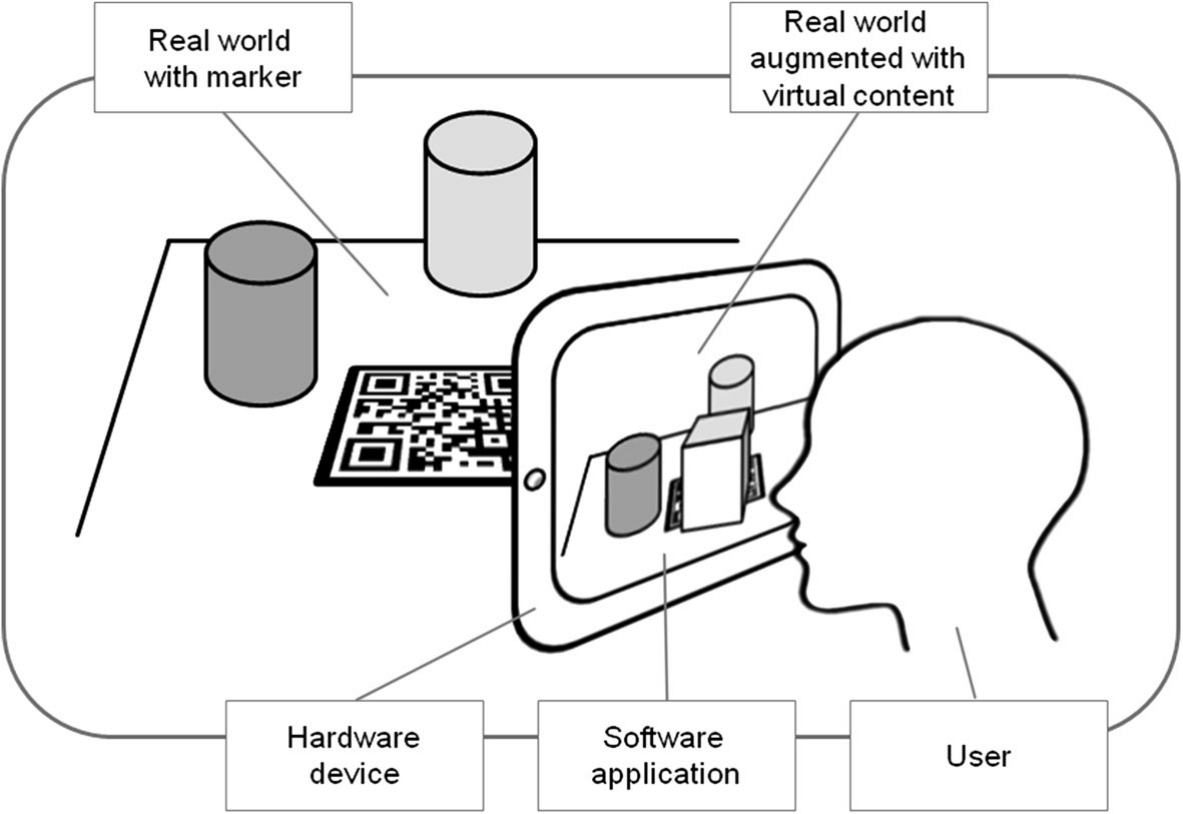
Virtual content (*block*) is added to the real world (*table*). A hardware device (*tablet*) including software is used to make the content visible for the user

In order to enrich the physical world with augmentations, a software application that uses one or more of the different hardware components must be installed on the device. There are two primary AR software implementation types: marker-based and markerless AR. Marker-based augmented reality uses 2D or 3D images such as a QR code (Fig. 1) or a physical object (for instance a building or humans [14]), which can be recognized by the software application. When the AR software application receives input from the marker or object, it generates the augmented virtual content and projects this information onto the recognized object. The user perceives that added information as really existing within the surroundings; he is immersed into an enhanced reality. Figure 1 clarifies this.

*Markerless augmented reality* uses positional data to acquire the user’s location with for instance a global positioning system (GPS) and a compass device to detect orientation [15] or with infrared light to create a depth image that produces data in the form of a silhouette (Kinect). Based upon this *tracking* information, the software application is able to augment the virtual content on a precise location on or within the real environment, regardless of whether or not that environment is static.

## Examples of AR systems for medical training

### Visualizing human anatomical structure with AR

Understanding human anatomy is essential for practising medicine since anatomical knowledge supports the formulation of a diagnosis and communication of that diagnosis to patient and colleagues [16]. Anatomy education is traditionally performed by the dissection of cadavers. ‘Anatomical dissection is the systematic exploration of a preserved human cadaver by the sequential division of tissue layers and the liberation of certain structures by removal of the regional fat and connective tissue with the aim of supporting the learning of gross anatomy by visual and tactile experience’ [17, p. 16]. The value of dissection classes as a teaching format lies in the fact that it provides a 3D view on human anatomy including tactile learning experiences. It enables elaboration of knowledge already acquired in lectures and study books and it provides an overall perspective of anatomical structures and their mutual relations in a whole organism [18]. This training format is, however, quite costly. And so far, no objective empirical evidence exists concerning the effectiveness of dissection classes for learning anatomy [17].

AR technology could offer an *additional* teaching method for anatomy education, depending on how it is implemented. Strong points are the visualization capabilities including the 3D rendering of anatomical imagery. Other sensory experiences could be implemented as well, such as tactile feedback. AR provides real-time manipulation of these visualizations and direct feedback to students. With that, AR technology could comply with some of the affordances of traditional dissection classes.

**Fig. 2:**
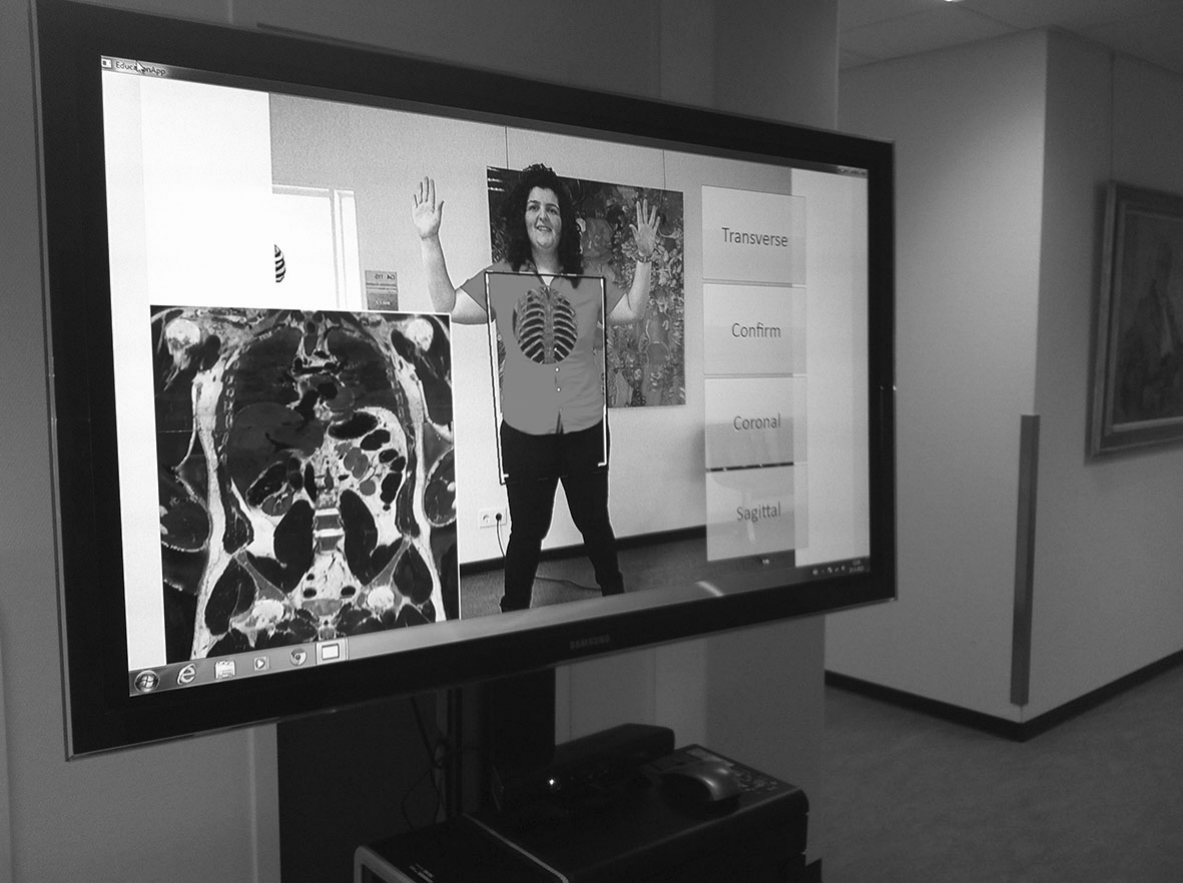
A screenshot of the magic mirror ‘Miracle’ that augments CT data onto the body of a trainee

Several AR systems have already been developed specifically for anatomy education [19–21]. Blum et al. [21] describe the magic mirror (‘Miracle’) which is an AR system that can be used for undergraduate anatomy education. The set-up of that system is as follows. The trainee stands in front of a TV screen that has a camera and the Kinect attached to it. The camera image of the trainee is flipped horizontally and shown on the TV screen, mimicking a mirror function (Fig. 2). Part of an anonymous CT dataset is augmented to the user’s body and shown on the TV screen. This creates the illusion that the trainee can look inside his body. A gesture-based user interface allows real time manipulation of the visualization of the CT data. The trainee can scroll through the dataset in sagittal, transverse and coronal slice mode, by using different hand gestures.

The Miracle offers several advantages for anatomy education depending on how it is embedded in the medical curriculum. It uses relatively inexpensive materials (Kinect, camera and TV) compared with the dissection materials. It provides a meaningful context (the whole human body) compared with textbook descriptions and stand-alone graphical presentations of anatomical structures (e.g. pictures, plasticized models). And it uses real-life material (the CT dataset) which can be applied in similar ways as in the professional context. This implementation of AR technology is promising because of its strong visualization and manipulation features. But, it does not yet do justice to the full potential of AR technology.

### Visualizing 3D lung dynamics with AR

AR also has great potential for visualizing more complex systems of the human body including the dynamic nature of such systems and patient-specific idiosyncrasies. Hamza-Lup et al. [22] developed a system that allows real-time visualization of 3D lung dynamics superimposed directly on a manikin or on a patient in the operating room. In that visualization they combine a generic functional lung model with patient-specific data extracted from high-resolution computed tomography (HRCT). This results in a dynamic, real-time visualization of virtual lungs that is overlaid onto the patient’s body. To see the 3D lung dynamics and possible deformations, the clinician wears a lightweight HMD.

This system can be used for specialist training trajectories during which various clinical scenarios can be trained. During non-invasive procedures, trainees can investigate the patient-specific breathing patterns and discomfort (e.g. dyspnoea) under different physical conditions and orientations of the patient in order to make accurate diagnostic decisions. Minimally invasive scenarios allow training of specific procedures such as intubation, endoscopy and needle insertion. More invasive scenarios such as lung transplants and lung volume reductions allow visualizing preoperative conditions and postoperative prognoses. This application of AR offers unique training opportunities that are only possible with this technology.

### Training laparoscopy skills with AR

The introduction of minimalistic invasive surgery (MIS) has given rise to a whole new surgical approach involving laparoscopy. Where open surgery has disadvantages for the patient, MIS is more demanding for the surgeon in terms of concentration, focused attention and the execution of complex psychomotor skills [23]. Laparoscopic procedures require the highly automated handling of laparoscopic instruments and being able to cope with the idiosyncrasies of this technique and its instruments. For instance, surgeons need to overcome the fulcrum effect. This refers to inversion and scaling of movement and altered sensations of force [24].

The laparoscopic psychomotor skill is a complex skill that can be unravelled into more fine-grained constituent skills. That is the production of motor actions and the recognition of environmental conditions that trigger those motor actions [25]. For example, a surgeon needs to be able to identify an anatomical structure at a certain location upon which he can start dissecting it with the laparoscopic instruments.

Motor abilities of expert surgeons show greater movement consistency and these experts are less fatigue prone. Expert surgeons also show excellent pattern recall and recognition when stimuli fit with an appropriate structure [26]. The latter includes the ability to envision depth and 3D, from 2D camera displays. An expert surgeon appears to execute both skills effortlessly and fluently.

Part-task practice is deemed the most effective training method for achieving excellent and automated mastery of such complex psychomotor skills [2, 25]. Part-task practice involves repeated and varied practice of a recurrent, constituent skill until a high level of automation is reached [2]. In the domain of laparoscopy training, many examples exist of AR applications that support such part-task practice with training scenarios that allow for training specific surgical procedures combining different motor abilities and anatomical structures to work on.

Botden and Jakimowicz [27] review four AR applications for laparoscopic surgery on their features, the extent to which empirical results support learning and their benefits. The reviewed systems are ProMIS, CELTS, Blue Dragon and LTS3e.

In these training environments, trainees train certain laparoscopic procedures with the same instruments as used in the OR. Basic recurrent skills are, for example, navigation with the trocars, and touching or grasping of tissue. More advanced recurrent skills that can be trained are transection or cutting, diathermia (heating of body tissue), dissection, or suturing. Trainees train on a manikin on which overlays of anatomical information are projected and the visual pathways of the laparoscopic instruments are shown. Sometimes the learning task is combined with a demonstration video and performance of the trainee is recorded.

The ProMIS system, for instance, combines a manikin with a laptop computer. Inside the manikin a tracking system measures the position and velocity of the surgical instruments. These data are subsequently visualized on screen.

Compared with real training environments (the so-called box trainers) and virtual reality training environments, the AR laparoscopy environments offer realistic haptic feedback which is essential for the transfer of laparoscopic skills to the work environment [28, 29]. In addition, these AR laparoscopy environments do not require an expert on-site to observe or guide the trainee.

These three examples of AR training systems are only a very limited selection of what is out there: how AR technology is used for medical training purposes. Yet, they already highlight the potential of this technology for learning and transfer. That potential is reflected in the use of the physical real-life context (or a context very similar to that), the advanced visualization capacity and simulation of other sensory information. And the training systems offer by large an active learning experience, in which interaction with the (real) world and direct feedback are paramount. Now that this potential merit is clear and we see the implementation of several dedicated training systems, a relevant question is what the empirical evidence for learning is.

## Empirical learning effects of AR training systems

When we look at empirical studies on learning effects supported by AR technology, a number of relevant types of research questions can be distinguished:

1. To what extent does an AR training system use a representative context, task, and behaviour compared with the real world? This is a matter of validity.
2. What learning effects does the AR training system generate?
3. What factors influence the implementation of an AR training system in a curriculum and how does that affect learning?
4. To what extent do learning results acquired with the AR training system transfer to the professional context?

Each of these research questions gives a relevant answer to the potential usefulness and effectiveness of AR supported learning. To our knowledge no empirical evidence has been published yet upon the effects of the Miracle for anatomy education. As for training with the dynamic 3D lungs, apart from assessing technical system behaviour, no empirical evidence on learning effects is known to us. Limited empirical evidence for AR laparoscopy training systems is available. Botden [29] for instance investigates ProMIS and assesses the extent to which the learning tasks in ProMIS sufficiently match the actual tasks to be performed during surgery. This is research that matches the first type of research question. The effect study reveals that both expert surgeons as well as surgeons-in-training judge the level of fidelity as sufficient and they estimate the didactic potential of ProMIS as a training tool to be large.

More research within the medical domain has been published on the effects of AR systems for learning but the results give a rather fragmented picture and no review studies have been performed yet. No firm conclusions can be drawn upon the established merit of AR for medical learning. Thus, we wonder what the status of AR for learning in other domains is and what is published in systematic reviews about that.

Schmitz et al. [30] present a review of practical research papers on augmented reality games for teaching and learning from a variety of domains. An augmented reality game combines mobile technology, gaming and geospecific activities with augmented content. The study results substantiate the motivational potential of augmented reality games and the potential to enhance knowledge acquisition. The limitations of this review are the fact that no established empirical results of AR are or could be reported. In addition, the augmented reality games all applied different game dynamics making an objective comparison difficult.

Wu et al. [7] performed a review of the literature about AR learning systems across domains and identified 54 published studies primarily in the fields of science and mathematics. These studies recognized learning benefits of AR systems specifically in the area of visualizing invisible or abstract concepts in order to promote conceptual understanding of dynamic models and complex causality. These studies also pointed out the motivational benefits of these systems and the role that immersion may play in that respect. This review did not include a systematic comparison of reported learning effects within different research designs.

Because no review studies have been done within the medical domain, there is a lack of a deep and systematic understanding of how AR can enhance complex learning in this domain. Also across domains no firm empirical results could be identified upon the effects of AR supported learning. We therefore suggest a systematic review of empirical research across domains on the characteristics of learning tasks in AR environments of interest to the medical domain and their established learning effects.

## Conclusion

The main question of this article was what AR is and what it could bring to the field of complex medical learning. We have seen that AR is the combination of a physical training environment that is very similar to or the same as the real-life environment augmented with visual and/or other sensory information. Learning supported with AR technology enables ubiquitous, collaborative and situated learning. It delivers a sense of presence, immediacy and immersion that may be beneficial to the learning process [7]. The affordances of such learning environments have the potential to stimulate meaningful learning, a necessary prerequisite for transfer of learning to occur. In the end, we of course aim for professionals who demonstrate excellence in the clinic.

Compared with studies of more mature educational technologies, many empirical studies upon the effects of AR (whether within the medical domain or outside) still focus on the development, usability and initial implementation of AR as a learning tool [7]. In order to establish the educational value of AR, the identified research questions need to be followed through with an adequate research design that includes large enough samples and valid measurements. Only then will the real merit of such advanced learning systems become clear. In that respect, we are on the eve of exploring the added value of AR for learning in the medical domain. Implementing such a novelty in the curriculum for medical professionals requires thoughtful development, its adoption only possible after empirical effect studies have proven the added value of AR for learning.

## Essentials

- In the medical domain, complex learning occurs that involves complex physiological systems, developing adaptive expertise and acquiring collaborative skills
- Learning in the medical domain is often situated in a real-life context. Training in this real-life context is not always possible
- AR learning environments potentially offer a meaningful situated learning experience that may enable transfer of learning into the workplace
- Up to now empirical research is performed primarily into the development, usability and initial implementation of AR learning environments. In that respect we are on the eve of exploring the added value of AR supported learning environments for learning
